# Data repurposing from digital home cage monitoring enlightens new perspectives on mouse motor behaviour and reduction principle

**DOI:** 10.1038/s41598-023-37464-8

**Published:** 2023-07-05

**Authors:** Sara Fuochi, Mara Rigamonti, Marcello Raspa, Ferdinando Scavizzi, Paolo de Girolamo, Livia D’Angelo

**Affiliations:** 1 Experimental Animal Center, University of Bern, Bern, Switzerland; 2 Tecniplast SpA, Buguggiate, Italy; 3 National Research Council, Institute of Biochemistry and Cell Biology (CNR-IBBC/EMMA/Infrafrontier/IMPC), International Campus ‘A. Buzzati-Traverso’, Monterotondo, Rome, Italy; 4 Department of Veterinary Medicine and Animal Production, University of Naples Federico II, Naples, Italy

**Keywords:** Neuroscience, Circadian regulation, Zoology, Animal behaviour

## Abstract

In this longitudinal study we compare between and within-strain variation in the home-cage spatial preference of three widely used and commercially available mice strains—C57BL/6NCrl, BALB/cAnNCrl and CRL:CD1(ICR)—starting from the first hour post cage-change until the next cage-change, for three consecutive intervals, to further profile the circadian home-cage behavioural phenotypes. Cage-change can be a stressful moment in the life of laboratory mice, since animals are disturbed during the sleeping hours and must then rapidly re-adapt to a pristine environment, leading to disruptions in normal motor patterns. The novelty of this study resides in characterizing new strain-specific biological phenomena, such as activity along the cage walls and frontality, using the vast data reserves generated by previous experimental data, thus introducing the potential and exploring the applicability of data repurposing to enhance Reduction principle when running in vivo studies. Our results, entirely obtained without the use of new animals, demonstrate that also when referring to space preference within the cage, C57BL/6NCrl has a high variability in the behavioural phenotypes from pre-puberty until early adulthood compared to BALB/cAnNCrl, which is confirmed to be socially disaggregated, and CRL:CD1(ICR) which is conversely highly active and socially aggregated. Our data also suggest that a strain-oriented approach is needed when defining frequency of cage-change as well as maximum allowed animal density, which should be revised, ideally under the EU regulatory framework as well, according to the physiological peculiarities of the strains, and always avoiding the “one size fits all” approach.

## Introduction

Profiling the motor behaviour of murine models has become one of the most widely used behavioural paradigms to determine the effects of various experimental approaches, e.g. genetic manipulation, pharmacological intervention, etc. Likewise other behaviours, differences in the motor activity between murine strains, which can be critical for in vivo research and influenced by the laboratory environment^1^, varies substantially across mouse strains^2^ and even substrains^3^.

The motor activity in mice, as in all mammals, is deeply influenced by the light exposure. As nocturnal animals, the peak of activity generally occurs during night hours while the light hours are spent for resting and sleeping. This biological trait is under the spotlight of the debate on translatability of murine models to human diurnal physiology. However, reversing light/dark cycles in the animal facility, which may provide an obvious way to study mice during their nocturnal active phase, may not represent a practical solution. Studying a nocturnal species at night is not the same as studying a diurnal species during the day, and adoption of such conditions must recognize these differences in temporal biology and consider the potential unintended consequences^4^.

The automated recording of motor behaviour of mice may represent an alternative and invaluable approach to overcome the translatability concern. Automated systems, which record the circadian in-cage mice activity, in absence of stress related to handling or behavioural apparatus, may provide unbiased observations which can be translated to circadian human physiology. Digital behavioural technologies have robustly confirmed the hypothesis that the basal diurnal activity of laboratory mice greatly varies among strains^5–8^, even when animals are kept under the same standardized husbandry conditions^3,7,9^.

A challenge in the field of automated assessment of behavioural phenotype is represented by the in-cage spatial pattern of diurnal motor activity. Automated analysis of behaviour has been reported in several experimental settings, such as in the classical anxiety-related behaviour, relying on patterns of movement near the wall and in the center of the cage^10^. However, to our knowledge, an automated analysis to define the circadian rhythmicity of the motor profiles of mice maintained under standard husbandry conditions has never been reported. In a previous study, we have characterized the night and day strain-specific activity of three non-genetically altered mouse strains, inbred (C57BL/6NCrl and BALB/cAnNCrl) and outbred (CRL:CD1(ICR)), through the analysis of different circadian metrics robustly demonstrating a clear strain-specific motor activity^5^. Here, we broaden knowledge on the spatial pattern of the motor activity. Interestingly, we aim to investigate and compare the in-cage spatial preferences of the three strains (C57BL/6NCrl, BALB/cAnNCrl) and (CRL:CD1(ICR)) following the diurnal activity and in response to the cage-change. The in-cage animal behaviour was longitudinally recorded 24 h/7 days by using Digital Ventilated Cages (DVC© Tecniplast S.p.A.) from the moment of the cage-change and repeatedly along three cage-change intervals. Cage-change, a routinely husbandry practice, is known to induce an alteration in the animal’s biologic equilibrium determining significant stress^11^. Although a routinary and necessary husbandry practice to keep mice and humans healthy, cage-change may be disruptive to mice, for several reasons, among which handling and manipulation, adapting to a new microenvironment and rebuilding the new cage ecosystem^12^ and likely modifying some sleep parameters^13^. Cage-change is known to influence the activity of these three strains, in terms of increasing the basal activity within a range of 5 h after the cage-change took place^5^, with strain specific features in terms of duration of the response to the cage-change and average activity recorded within the estimated response.

In this study we introduce new spatial measures to longitudinally disentangle the spatial patterns of motor activity of the three strains in response to cage-change, scheduled every second week, and in-between three cage-change intervals. Spatial measures refer to the (i) activity along the cage walls, calculated as the percentage of activity performed over the eight lateral electrodes of the cage and (ii) frontality, calculated as the percentage of activity performed in the front of the cage^10^. In addition to these spatial measures, we introduced the Gini index (or Gini coefficient), a statistical measure, widely used in economics to describe the (in)equality of the distribution of wealth or income between individuals in a population^14^. The use of Gini index is being nowadays used also in life sciences to identify, for instance, those genes whose expression varied least across a large set of samples^15^. In this study, we used the Gini index to understand if the activity is distributed over all the cage floor or concentrated in a smaller area. Remarkably, the longitudinal analysis of these measures led us to correlate spatial preferences of mice from pre-puberty until early adulthood, generating an accurate reconstruction of the temporo-spatial pattern of spontaneous motor activity of the three strains in a very delicate phase of the animal life cycle.

Most interestingly, these new developed measures were calculated from data obtained by the previous recordings^5^, in full compliance with the concept of reducing the number of animals used in experimental settings. The striking potential of using advanced technologies, such as DVC® systems, relies also on the possibility to re-purpose archived data to introduce new information which contribute to portray, in an unbiased approach, the spontaneous motor activity of the experimental animals.

## Results

Thanks to the data recorded by the DVC® system on the monitoring of movement of three commonly used mouse strains (C57BL/6NCrl, BALB/cAnNCrl and CRL:CD1(ICR)), we reconstruct the spatio-temporal pattern of spontaneous motor behaviour of group housed animals from 5 to 12 weeks of age, covering the period between pre-puberty, sexual maturity and early adulthood. We compared male and female mice of the three strains, BALB/cAnNCrl, C57BL/6NCRL and CRL:CD1(ICR), over a period of about 2 months, starting from the first hour post cage-change until the successive, for three consecutive cage-change intervals. The strain C57BL/6NCRL was used as reference strain, as in the previous study^5^.

### First hour post cage-change

**Figure 1:**
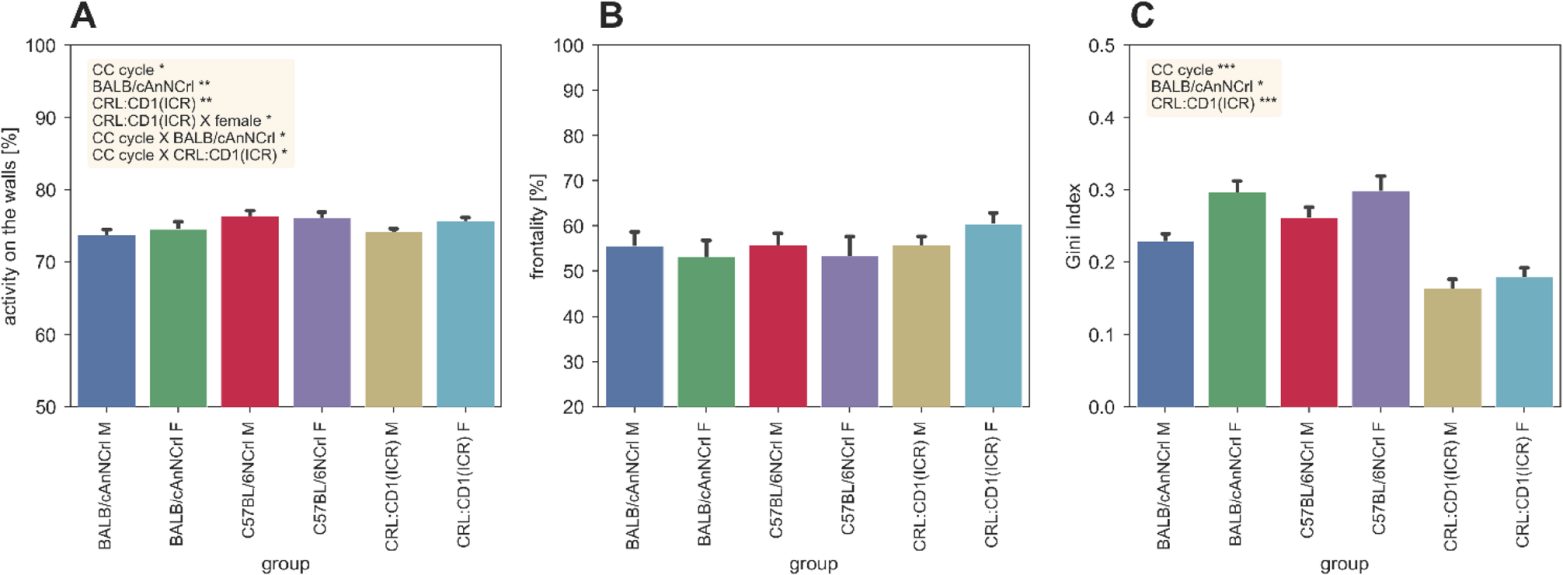
Spatial distribution metrics across the first hour of cage-change. (**A**) Average (± s.e.m.) percentage of activity performed over the 8 lateral left and right electrodes with respect to the total activity recorded over all the 12 electrodes. (**B**) Average (± s.e.m.) percentage of activity performed over the 6 frontal electrodes with respect to the total activity recorded over all the 12 electrodes. (**C**) Average (± s.e.m.) Gini Index calculated over the activity values of the 12 electrodes. In each panel, significant fixed effects are reported (*p < 0.05, **p < 0.01, ***p < 0.001), with male C57BL/6NCRL used as reference (all model structures and relative statistical results are available in supplementary data materials).

Within the first our post cage-change, our model (Supplementary Information) revealed that the percentage of activity along the cage walls was overall lower in the BALB/cAnNCrl (p_BALB/cAnNCrl_ < 0.01) and CRL:CD1(ICR) (p_CRL:CD1(ICR)_ < 0.01) mice strains compared to C57BL/6NCrl, with cages of CRL:CD1(ICR) females significantly higher compared to CRL:CD1(ICR) males (p_CRL:CD1(ICR):female_ < 0.05) (Fig. 1A). On the opposite, the percentage of motor activity spent in the frontal part of the cage was comparable in both sexes of the three strains (Fig. 1B). This pattern was confirmed when comparing all cage-change intervals. We then analysed the spatial distribution of activity of group-housed mice within each single cage by calculating the Gini Index (Fig. 1C), and very interestingly we observed that CRL:CD1(ICR) (p_CRL:CD1(ICR):Gini index_ < 0.001) and, to a less extent, BALB/cAnNCRL (p_BALB/CANNCR:Gini index_ < 0.05) occupied more cage floor than C57BL/6NCrl (Fig. 1C), even if our model displayed slight differences over the cage-change intervals (p_CC-cycle:Gini index_ < 0.001). Coherently, the analysis of variance confirmed the wider distribution of CRL:CD1(ICR) (p_CRL:CD1(ICR):variance_ < 0.01) in the whole cage compared to the other two mice strains (Supplementary Information).

### Day and night activity after cage-change and in-between cage-change interval

**Figure 2:**
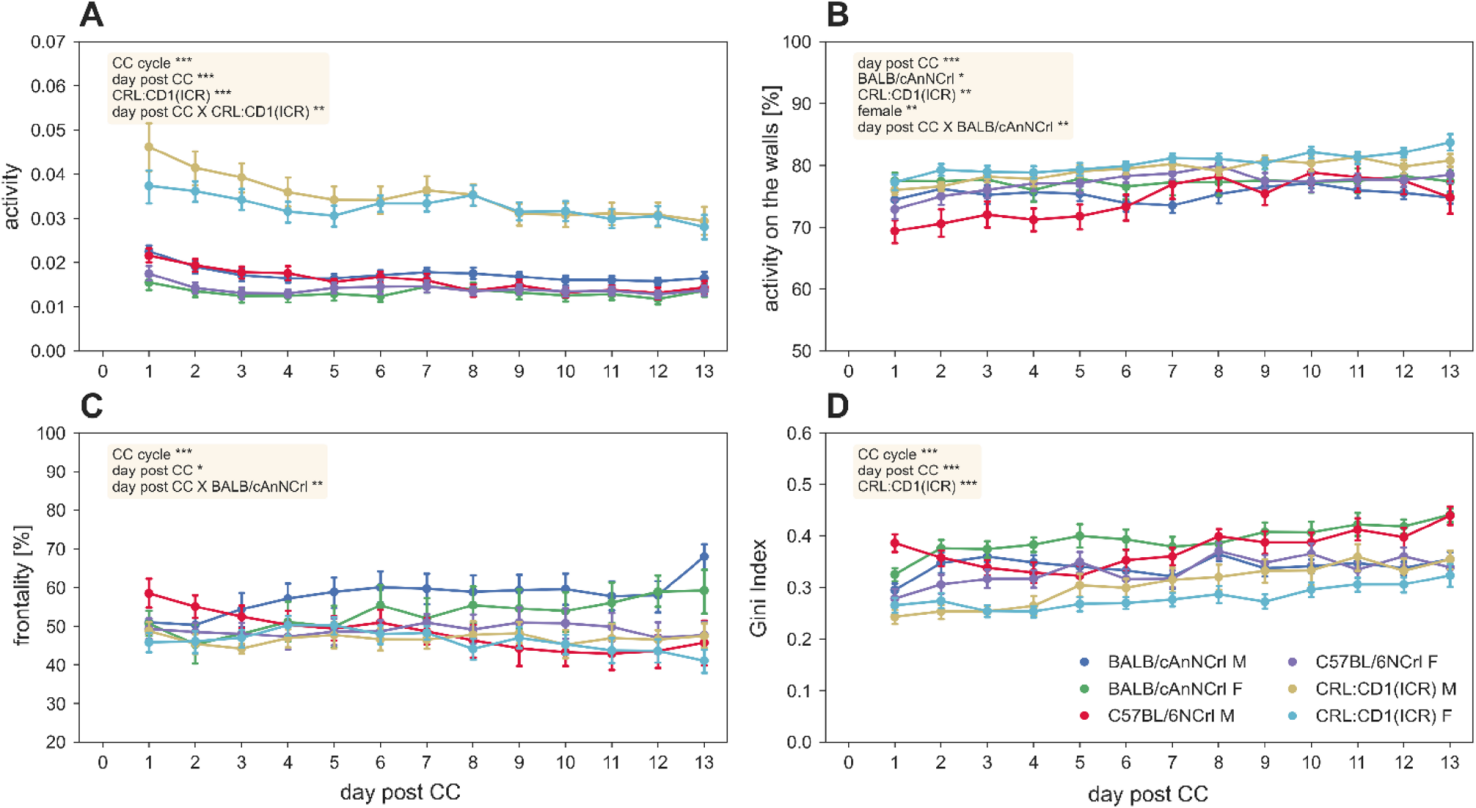
Activity metrics during the lights-on phase. (**A**) Average (± s.e.m.) activity. (**B**) Average (± s.e.m.) percentage of activity performed over the walls. (**C**) Average (± s.e.m.) Frontality. (**D**) Average (± s.e.m.) Gini Index of the activity values of the 12 electrodes. The metrics are expressed over the days of cage-change cycle (cage-change day excluded). In each panel, significant fixed effects are reported (*p < 0.05, **p < 0.01, ***p < 0.001), with male C57BL/6NCRL used as reference (all model structures and relative statistical results are available in supplementary data materials).

The spontaneous motor activity during the light and dark hours after cage-change and in-between cage-change intervals (of 14 days) was analysed, and, consistently with our previous data^3^, the activity of either males or females of CRL:CD1(ICR) during light hours was more intense (p_CRL:CD1(ICR):activity:light phase_ < 0.001) than that displayed by the two inbred strains (Fig. 2A).

Interestingly, during the light phase CRL:CD1(ICR) and BALB/cAnNCRL performed most of their activity close to the walls, with the highest significance observed the first day post cage-change (p_CRL:CD1(ICR):walls percentage:light phase_ < 0.01 and p_BALB/cAnNCrl:walls percentage:light phase_ < 0.05), compared to C57BL/6NCrl mice (Fig. 2B). This motor spatial pattern was more evident in females of the two strains (p_female:light phase_ < 0.01). Furthermore, BALB/cAnNCRL displayed an increase of activity in the frontal part of the cage over time (p_BALB/cAnNCrl*day_post_CC:frontality:light phase_ < 0.01) (Fig. 2C). During the light phase, the recording of spontaneous activity of CRL:CD1(ICR) confirmed a wider distribution in the whole cage differently from the two inbred strains (p_CRL:CD1(ICR):Gini index:light phase_ < 0.001) (Fig. 2D). Spatial average activity heatmaps are shown in the Supplementary Information.

**Figure 3:**
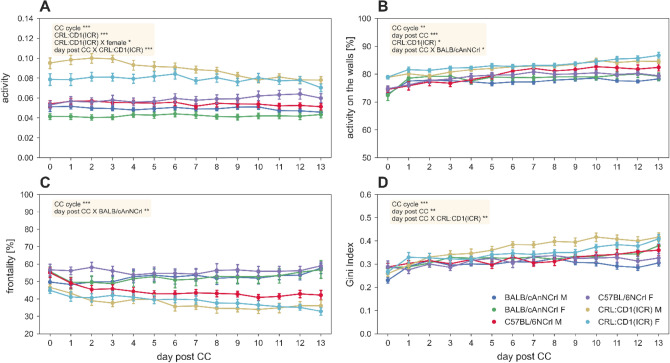
Activity metrics during the lights-off phase. (**A**) Average (± s.e.m.) activity. (**B**) Average (± s.e.m.) percentage of activity performed over the walls. (**C**) Average (± s.e.m.) Frontality. (**D**) Average (± s.e.m.) Gini Index of the activity values of the 12 electrodes. The metrics are expressed over the days of cage-change cycle. In each panel, significant fixed effects are reported (*p < 0.05, **p < 0.01, ***p < 0.001), with male C57BL/6NCRL used as reference (all model structures and relative statistical results are available in supplementary data materials).

During the dark phase, CRL:CD1(ICR) was still the most active strain (p_CRL:CD1(ICR):activity:dark phase_ < 0.001) with a reduction over time for all the strains (p_CRL:CD1(ICR):day_post_CC:dark phase_ < 0.001) (Fig. 3A). Only CRL:CD1(ICR) performed most of their activity along the cage walls (p_CRL:CD1(ICR):walls percentage:dark phase_ < 0.05) compared to the inbred strains (Fig. 3B), while the activity in the frontal part of the cage was overall consistent with the data observed during the light phase (Fig. 3C). Very interestingly, since the first day post cage-change and over days until next cage-change, CRL:CD1(ICR) were mostly active on few electrodes (p_CRL:CD1(ICR)*day_post_CC:Gini-index:dark phase_ < 0.01) (Fig. 3D). This pattern was also observed over all analysed cage-change intervals, even with slight differences (p_CC-cycle:dark phase_ < 0.01). Spatial average activity heatmaps are shown in the Supplementary Information.

### Spatial pattern of 24-h motor activity in-between the cage-change interval

**Figure 4:**
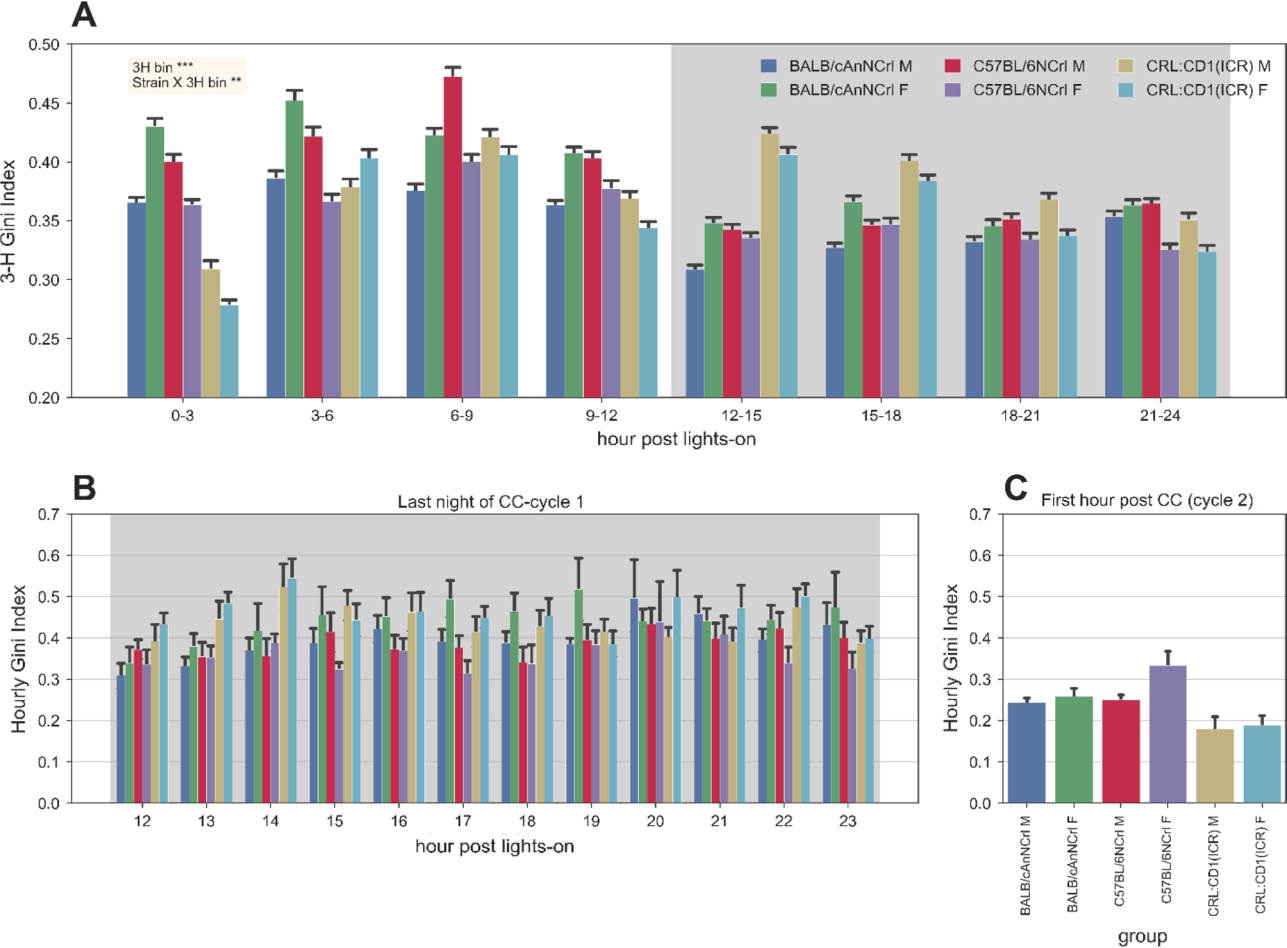
Gini Index in-between the cage-change interval. (**A**) Average (± s.e.m.) Gini Index of activity values of the 12 electrodes calculated for each 3-h bin across all days and cage-change cycles. Significant factors from ATS nparLD test are reported (**p < 0.01, ***p < 0.001). (**B**) Average (± s.e.m.) Gini Index of activity values of the 12 electrodes calculated for each hour of the night previous the cage-change, during the first cage-change cycle (example, see all the data in the Supplementary Information). (**C**) Average (± s.e.m.) Gini Index of activity values of the 12 electrodes of the first hour of the second cage-change, following the night in 4B (example, see all the data in the Supplementary Information).

In the previous study, we analysed the pattern of 24-h motor activity of the three strains documenting that CRL:CD1(ICR) were overall more active than the other two ^5^, also during the light phase (Fig. 4A). Here, we document that the spatial pattern of the motor activity changes during the day (p_bin_3hs:hourlyGini index_ < 0.001), especially when comparing light and dark phase. During the first three hours of light phase, CRL:CD1(ICR) activated all electrodes confirming that the activity was wider distributed over the entire cage. Very surprisingly, during the dark phase the same groups displayed bouts of activity in a restricted area of the cage (likely corresponding to two electrodes). On the opposite, C57BL/6NCrl, and to a less extent, BALB/cAnNCrl, spent their activity in a smaller area during the light phase compared to the dark phase.

These observations prompted us to analysed more in-depth the spatial pattern of the three strains. At this aim we visualized the last twelve night-hours before cage-change with the first hour after the following cage-change, to highlight any behaviour variation with regards to the highest and lowest level of habituation of animals to the cage environment. CRL:CD1(ICR) performed the activity in the whole cage in the first hour post cage-change, while over days the activity was recorded in a small area of the cage, in correspondence of only two electrodes (Fig. 4B,C). These observations were confirmed for all cage-change intervals (see Supplementary Information).

### Correlation between activity and latrine distribution

**Figure 5:**
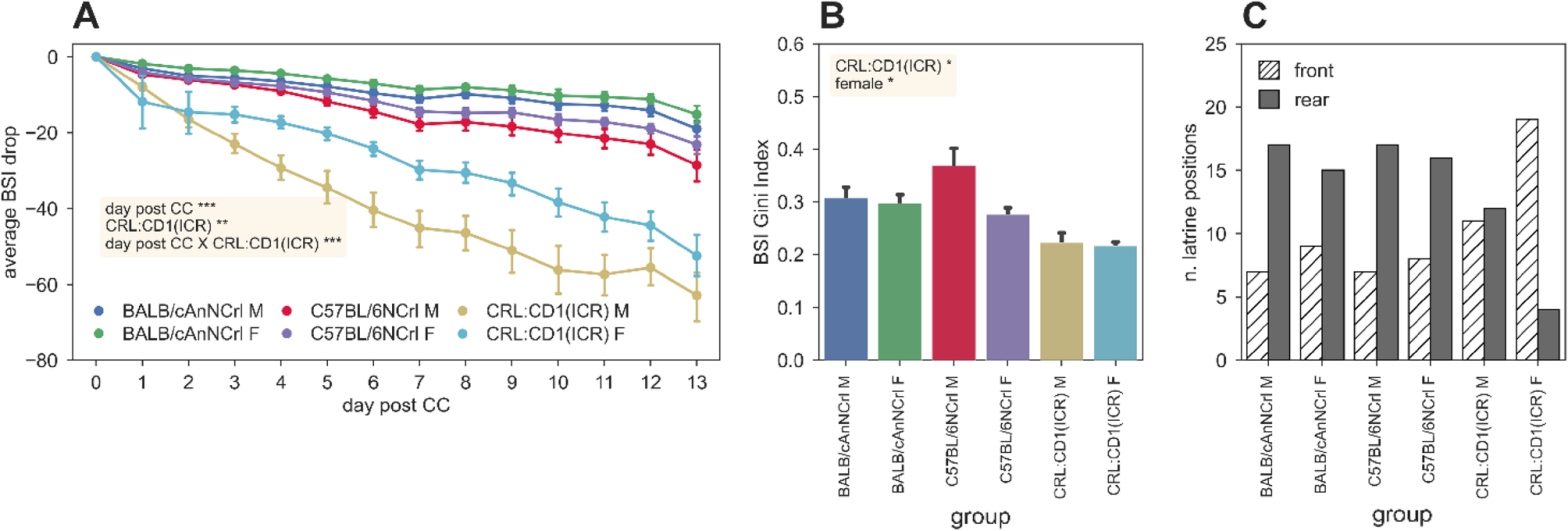
Latrine metrics and spatial distribution. (**A**) Average (± s.e.m.) Bedding Status Index difference between each night and the first night of the corresponding cage-change cycle. (**B**) Average (± s.e.m.) Gini Index of the BSI difference values of the 12 electrodes on the last night of each cage-change cycle. (**C**) Position of the latrine determined by DVC, across all the cage-change cycles. In panel A and B, significant fixed effects are reported (*p < 0.05, **p < 0.01, ***p < 0.001), with male C57BL/6NCRL used as reference (all model structures and relative statistical results are available in supplementary data materials).

Since the DVC® system allows to evaluate the bedding status by measuring the average signal drop^9^, we hypothesized that the latrine position could be among the factors influencing the animal activity. Indeed, it is well demonstrated that mice can segregate space into clean and dirty areas^16^. In the timeframe between the last and the first night of the cage-change interval, for each electrode the average signal drop of the bedding was higher in the cages of CRL:CD1(ICR) (p_CRL:CD1(ICR):BSI-drop_ < 0.01), and more widespread (p_CRL:CD1(ICR):Gini index:bedding status_ < 0.05) compared to the cages housing the other strains (Fig. 5A,B). Differently, in cages of males, the average drop was more concentrated on few electrodes (p_female:Gini index:bedding status:female_ < 0.05), especially in C57BL/6NCrl. In agreement with this observation, there is a significant negative Pearson correlation of Gini Indices of activity and BSI drop in the dark phase (r = − 0.28, *p* < 0.01) and a positive correlation during light (r = 0.30, *p* < 0.01) phase. These data were also confirmed by the spearman correlation (rank-based) (r_dark phase_ = − 0.24, *p* < 0.01; r_light phase_ = 0.35, *p* < 0.01). Finally, we could precisely identify where animals prefer to perform their activity based on the decision on the placement of the latrine. This latter was localized more in the rear of the cages of C57BL/6NCrl and BALB/cAnNCrl males, while more in the frontal part of the cages of CRL:CD1(ICR) females (Fig. 5C).

## Discussion

The investigation and characterization of mice behaviour remain a fundamental part of the analysis of many biological systems and, therefore, are of great interest to the in vivo research. Traditionally, motor behaviour has been analyzed as an indicator of the effect or function of drugs, genes, and disease models^17^. In the last decades the scientific community has largely recognized the relevance as well as the impact of some factors (i.e. light, feed, transportation, cage-change, etc.) related to the environment, on the animal motor activity and thus on animal behaviour and welfare^18^. Here, for the first time we longitudinally delineate the circadian profiles of motor behaviour in response to the cage-change of male and female group-housed mice.

The novelty of this study resides in characterizing new biological phenomena, such as the spatial pattern of spontaneous motor activity of three mice strains, within the vast data reserves generated by previous experimental data. We thus introduce the concept of data repurposing in an in vivo study. *Data repurposing* refers to the re-use of data for a completely different decision/task to what it was originally intended to be used for^19^. It has now become an increasingly acknowledged method to re-analysing original data sets using alternate or integrative methods to answers questions that could not be seen, answered or fully understood with any of the original data processing. It is crucial, then, to consider that in life science too^20^, one of the roles of big data and data analysis might be to provide progressively closer look to old dataset and set the stage for a re-analysis of the same data, at some later stages, to improve understanding of the data, and answer questions that were not posed during the first analysis^21^. Data repurposing must find its own use and publishing etiquette, under the ethical and practical perspective in the life-sciences field. Consistently with Norman and Griffiths^22^, in this work, to secure ethical integrity, we extensively refer to the previous work on spontaneous motor activity, and proactively bridge old and new analysis to highlight distinction in analysis tool and further interpretation of the data, fully compliant with the 3Rs principle as well as ensuring reliable and reproducible in vivo experimental data^7,9^.

We compared between and within-strain variation in the home-cage spatial preferences of the three mice strains, starting from the first hour post cage-change until the successive cage-change, for three consecutive intervals to profile the circadian home-cage behavioural phenotypes. Cage-change is a stressful moment in the mouse cage life since animals are disturbed during the sleeping hours and must adapt to the “new” environment^11,23^. We observed that within the first hour post cage-change, CRL:CD1(ICR) and BALB/cAnNCRL displayed bouts of activity over the whole cage floor, while C57BL/6NCrl moved more along the cage walls. The pattern was inverted from the day after cage-change and over the whole cage-change interval. During the lights-on phase, CRL:CD1(ICR) and BALB/cAnNCRL performed most of their activity along the walls than in the center of the cage, with the highest fraction of activity recorded for the animals of the outbred strain moving along the whole cage perimeter. Most remarkably, during the lights-off phase the pattern of CRL:CD1(ICR), more active and dynamic than the two inbred strains^5^, performed the mostly of their activity on only two electrodes. Further analyses of the circadian pattern of spatial motor activity over the cage-change intervals confirmed these observations, corroborating the hypothesis that this strain is highly active in a narrow area. We attributed this peculiar pattern to the mouse capability to segregate activity and resting areas from areas where they defaecate and urinate^16^. Although in our experiments animals were housed in a standard cage, where feces are dispersed throughout all locations, likely because of mixing and moving of bedding during activity making thus difficult a physical separation from the nesting activity space^16^, the evolutionary conserved behaviour to remain in a cleaned environment was distinctly captured by the DVC® system. This segregation was evident also for the other two strains, which mostly occupied the area opposite to the latrine position. Specifically in the case of CRL:CD1(ICR), whose intense activity was confined in a very narrow area of the cage, and in close proximity of the fresh-air inlet, the larger amount of waste products, likely due to the larger size of the outbred strain, negatively impacts the bedding status thus suggesting that the husbandry practice of cage-change should be revised according to the physiological needs of each mouse strain. Defining the ideal cage change interval for a specific strain, as well as reliable parameters to use to assess animal wellbeing with regards to cage environment is not trivial.

An interesting study from Vogelweid et al.^36^ suggested that based on ammonia levels in the cage and histopathological correlated lesions, a 2-weeks cage change interval could appropriate for CRL:CD1(ICR) housed in groups of 3. Interestingly, authors stated that human perception of cage cleanliness was not a reliable predictor of air quality within the cage and that mice with rhinitis could not be reliably identified by visual observation.

Here, we would like to move forward, adding clear behavioural, strain specific patterns that could improve the understanding of cage quality and animals interaction with the cage environment. Thanks to the DVC, we were able to observe how CRL:CD1(ICR) mice quickly confined their activity in a very narrow area of the cage, while latrine area expands in the cage. It is also remarkable that the areas of confined activity are in close proximity of the fresh air inlet. In our opinion, these observations suggest how CRL:CD1(ICR) mice housed in standard individual ventilated cages may fail in the attempt to segregate activity and nesting sites from elimination sites. This, due to their bigger size and higher urine and feces output when compared to analyzed inbred strains, as well as to their highest levels of activity which could enhance the spreading of urine and feces around the cage^16^. Assessing weather increasing cage change frequency vs housing in sub-optimal cage environment would negatively affect the wellbeing of animals may require a case by case harm/benefit analysis.

To this purpose, other preventive measures rather than simply increasing cage change frequency should be considered, and further consideration should be given to cage density, particularly when considering CRL:CD1(ICR) mice, which can easily reach more than 30 g in 7 weeks old females, and more than 40 g in 10 weeks old males^24^. Remarkably, the Directive 2010/63/EU of the European Parliament and of the Council of 22 September 2010 on the protection of animals used for scientific purposes, guarantees a progressively increasing surface to mice based on 5 g increase in the range of 20 to 30 g, and requires a minimum of 100 cm^2^ of surface to mice weighing any weight above 30 g, thus allowing up to 5 animals in this open range in a standard 535 squared centimeters cage. Therefore, under the Directive 2010/63/EU, in example, up to 5 CRL:CD1(ICR) weighing more than 30 g could be co-housed in a standard 535 squared centimeters cage.

In the light of the dramatic difference of bedding status index between CRL:CD1(ICR) vs C57BL/6NCrl and BALB/cAnNCRL mice, and specific behavioural patterns showed by CRL:CD1(ICR), further and more granular weight ranges/surface/density provisions should be considered by Regulatory authorities to secure minimal hygienic conditions and welfare, by reducing the maximum number of animals allowed per standard 535 squared centimeters cage, by increasing the minimum surface requirements for specific, demanding strains.

The two inbred strains displayed overall comparable spatial pattern of spontaneous motion, accordingly to previous reports using different experimental approaches^25,26^. However, some strain-specific traits were noted: the motor activity of C57BL/6NCrl was more variable over the cage-change intervals than the other two strains. When animals were introduced in the new cage, both males and females performed more activity along the cage walls compared to the other two strains, consistently with previous documented behavioural observations on this specific substrain^3^. Remarkably, the motor activity in all cages of C57BL/6NCrl changed in response to the cage-change over the two months. Over time, animals performed most of their diurnal activity on a wider surface of the cage floor. On the opposite, over the experimental observations increasing activity was recorded in the frontal part of the cages of BALB/cAnNCRL. Most remarkably, our data document that BALB/cAnNCRL mice activated all electrodes, suggesting thus that they perform their activity without social conspecific interactions, accordingly to the behavioural feature of low sociability, already well characterized^27^.

Our longitudinal analyses enabled us also to correlate the spatial pattern of motor behaviour with the age of animals, which is known to vary across lifespan^28^. The spontaneous motor activity of the three strains was recorded from pre-puberty until early adulthood of animals. These phases are critical in the lifetime of mice being characterized by a multitude of hormonal and behavioural changes as well as remodeling of neuroanatomical structures, leading to cognitive, emotional, social, and sexual maturation^29^. Generally, the phase of adolescence is characterized by behaviour and physiology that differs substantially from adulthood, *e.g.* there is a general trend of increased open field activity throughout all three stages of adolescence into adulthood^28^. Adolescence-related motor behaviour in open-field test appeared more explorative and less anxious in CRL:CD1(ICR) animals of both sexes, compared to adult animals^30^, while activity in C57BL/6J strain increased from the early and mid-adolescent phase to adulthood^31^. Our findings document a more variable motor behaviour in the C57BL/6NCrl strain over the two months, and a steadier and more constant pattern of spontaneous motor activity in the other two strains. Key differences in C57BL/6NCrl were observed in response to the cage-change: in the early adolescence (starting at weaning and characterized by the onset of sexual maturation^28^) we observed more pronounced thigmotaxis within the first hour post cage-change, which appeared less evident in the late adolescence, when animals were distributed over the entire cage floor. Conversely, strictly referring to the effect of cage-change, performed during the light phase, BALB/cAnNCRL and CRL:CD1(ICR) displayed a strain-specific but well conserved spatial behaviour from pre-puberty to early adulthood, maintaining a smaller activity on the walls. Collectively, these data may suggest a distinct feature of the physiological light responses of albino mice, particularly since such similarities are transversal between an inbred and an outbred albino strain. Nonetheless, based on our results and considered the strains analyzed so far, we are unable to demonstrate clear correlation between motor behaviour and space preference considering albinism *versus* pigmentation. Further variables might have played a role, including type of light until weaning and developmental phase at our first experimental conditions exposure^32^ as well as composite genetic component and potential multigenic impact^33^ for which a direct comparison between C57BL6/N and C57BL6/N albino would provide a more reliable experimental design. We are planning more tailored experimental approaches aiming at comparing the spontaneous circadian activity in albino and pigmented mice to better explore the effects of light on mouse motor behaviour.

In conclusion, this study witnesses how the vast amount of data generated by digital technologies may robustly contribute to the implementation of the “R” of Reduction in in vivo research and identify new biological data. However, a rigorous approach must be maintained when dealing with the re-purposing of data, also due to ethical implications. This approach has enabled us to characterize further behavioural phenotypic traits of the three strains, useful for future comparative studies. We demonstrated that C57BL/6NCrl strain has a high variability in the behavioural phenotypes from pre-puberty until early adulthood compared to BALB/cAnNCrl, which is the least active and socially disaggregated and CRL:CD1(ICR), the highest active and socially aggregated. Thanks to this in-depth characterization, we could draw meaningful conclusions about the spatial preference of CRL:CD1(ICR). The highest activity recorded on only two electrodes, correlated to the Bedding Status Index, supports also a practical consideration on the frequency of mice cage-change as well as animal density from this strain. Density and frequency of cage-change should be based on the physiological needs of the specific strain, confirming that in life sciences there is no “one size fits all” approach.

## Methods

### Ethical approval

All methods were carried out in accordance with relevant guidelines and regulations and are reported in accordance with ARRIVE guidelines. Data presented here entirely derive from re-analysis and re-purposing of previously recorded data^5^. Therefore, no new animals were used to perform this study. The study, data collection and analysis were approved by the Institutional Animal Care and Welfare Body of the CNR-IBBC/EMMA/Infrafrontier. Animal maintenance was performed in accordance with general guidelines regarding animal breeding and biotechnology, in compliance with the Italian Legislative Decree 26/2014.

### Mice

The in-cage spontaneous motor activity of C57BL/6NCrl, BALB/cAnNCrl and CRL:CD1(ICR), commercially available from Charles River Laboratories was previously recorded^5^. The mice were bred under barriered specific pathogen free–condition facilities at the Charles River Laboratory facility in Calco, Italy according to internal breeding standard operating procedures, which include a genetic stability program and specific pathogen free conditions. At 3 weeks of age, after weaning, the mice were moved to the CNR-IBBC/EMMA-Infrafrontier-IMPC Core Structure (Monterotondo, Rome, Italy)—Consiglio Nazionale delle Ricerche (Italy) and housed in DVC® racks for the whole duration of the study. After acclimatization, mice of each strain were housed in groups of three individuals per cage, fed ad libitum with standard diet (4RF21; Mucedola), under standard controlled environmental parameters (temperature = 21 ± 2 °C; relative humidity = 55% ± 15%), and mice were kept in a 12-h light/12-h dark cycle (7 AM–7 PM: lights on) with 12–15 air changes per hour and a 12:12 light cycle. Light intensity at room level was 230 lx, while cages were exposed to slight differences according to their position within the rack. Variations of light intensity at cage level were recorded, with lux levels ranging from 29 to 12 lx. Certified dust-free wood bedding (Scobis one; Mucedola) was provided in the cages. Mice were provided chlorinated, filtered water ad libitum. 2-week-interval cage-changes were adopted with unaltered standard procedure and timing (Mondays 9 AM–3 PM—light phase). To allow for cage-change, animals were shortly restrained by tail base, and moved to the new cage. Paper nesting material was provided as environmental enrichment. Cage density was standardized to three mice per cage, with the intent to mimic possible standard housing conditions in research settings, avoiding the potential bias provided in terms of motor activity by single housing (i.e., absence of interaction with cage mates and altered (increased) time to integrate into the nest, leading to a prolonged activity time^34^.

Experimental groups were divided in two separate cohorts of mice in two different periods of the year (springtime and late summer/early autumn) to reduce the seasonality bias, as follows: C57BL/6NCrl mice, *n* = 18 males (6 cages); *n* = 18 females (6 cages); BALB/cAnNCrl mice, *n* = 18 males (6 cages); *n* = 18 females (6 cages); CRL:CD1(ICR) mice, *n* = 18 males (6 cages); *n* = 18 females (6 cages). Each cohort was thus composed of 54 individuals (27 females plus 27 males equally divided per strain).

### Home-cage activity monitoring: DVC® system and metrics

**Figure 6:**
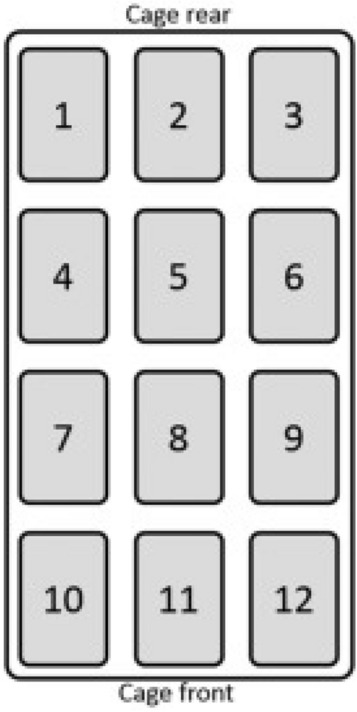
Electrodes of the DVC board. Position and numbering of the 12 electrodes of the DVC board.

The DVC® rack is a home-cage monitoring system that automatically measures animal activity 24/7. An electronic capacitance sensing board is positioned below each cage and consists of 12 contactless electrodes that record the animal’s presence in each electrode surrounding (Fig. 6). We used the Animal Locomotion Index Smoothed (DVC Analytics, Tecniplast S.p.a.), which is based on activation density^35^ to capture mouse activity in the cage, and we aggregated it in light and dark phases. To study the spatial distribution of the activity over the cage floor, we calculated Frontality, which is the percentage of activity performed over the six electrodes of the frontal part of the cage (7, 8, 9, 10, 11, 12) over the total activity across all the cage floor^9^ and the activity along the cage walls, calculated as the percentage of activity performed over the four electrodes on the left side (1, 4, 7, 10) and the four electrodes on the right side (3. 6, 9, 12). The choice of the activity along the walls metric, was done in an effort to detect thigmotactic-like behaviors, since trajectories were not an option, as animals were group housed. We also used the frontality metric as it already proved to be a reliable one, to assess the spatial distribution of activity over time (9). We then decided to calculate the Gini Index of the activity values of the 12 electrodes, to capture how the activity is distributed over all the 12 electrodes. Measuring the inequality of a distribution, low levels of Gini Index indicate that the activity is quite homogeneous over the whole cage floor, with 0 corresponding to same activity levels for all the 12 electrodes, while high levels of Gini Index indicate that most of the activity is concentrated on few electrodes, with a Gini Index of 1 when the 100% of activity is performed over just one electrode.

Since the capacitance measured by the DVC® board is affected by the humidity inside the cage and therefore urine^35^, we also determined the position of the latrine^7^ and its evolution over time by using the Bedding Status Index (BSI; DVC Analytics, Tecniplast S.p.a.), averaged across each night and compared with respect to the first night of the corresponding cage-change cycle. We then calculated the Gini Index of the BSI values of the 12 electrodes to measure the dispersion of the latrine over the cage floor.

### Statistical tests

Because the same individuals were assessed over time and for a long period (60 d), we used general linear mixed models to quantitatively evaluate differences between strains, sexes and time and light conditions. We used lmerTest R software package to model data and test for fixed effects. We resorted to a top-down approach and successive likelihood ratio tests to define the model best explaining the data. All selected models and relative statistical results are available in supplementary data materials. We also used a rank-based analysis of variance-type statistic (ATS, as implemented in the nparLD R package) to test differences across different 3-h bins during the 24H day. We used Python to process and visualize data and R (version 3.4.3) to run all statistics, with significance level α = 0.05. We conducted calculation on three cage-change intervals, by excluding i. the first interval, corresponding to acclimation of animals; ii. the days of cage changing from the daily analysis; iii. the days with missing values or with some technical issues. As a consequence of group housing, the statistical unit is the cage: DVC® measures the overall aggregated value of activity of the mice for each cage, with a reduction of statistical power that is not necessary scaled down exactly with the aggregation factor, because of probable intra-cage correlation.

## Supplementary Information

Supplementary Information 1.

Supplementary Information 2.

## Data Availability

The datasets used and/or analysed during the current study available from the corresponding author on reasonable request.
