# Supplementary material for: Data repurposing from digital home cage monitoring enlightens new perspectives on mouse motor behaviour and reduction principle
Source: Sci Rep. 2023 Jul 5;13:10851. doi: 10.1038/s41598-023-37464-8 (PMC10322864; doi:10.1038/s41598-023-37464-8)
Supplement: Supplementary file 1 — Supplementary Information 1. [file 41598_2023_37464_MOESM1_ESM.docx]

**Data repurposing from digital home cage monitoring enlightens new perspectives on mouse motor behaviour and reduction principle**

**Supplementary Information**

Availability of Data and Materials

The datasets used and/or analysed during the current study available from the corresponding author on reasonable request.

**Supplementary Figures**


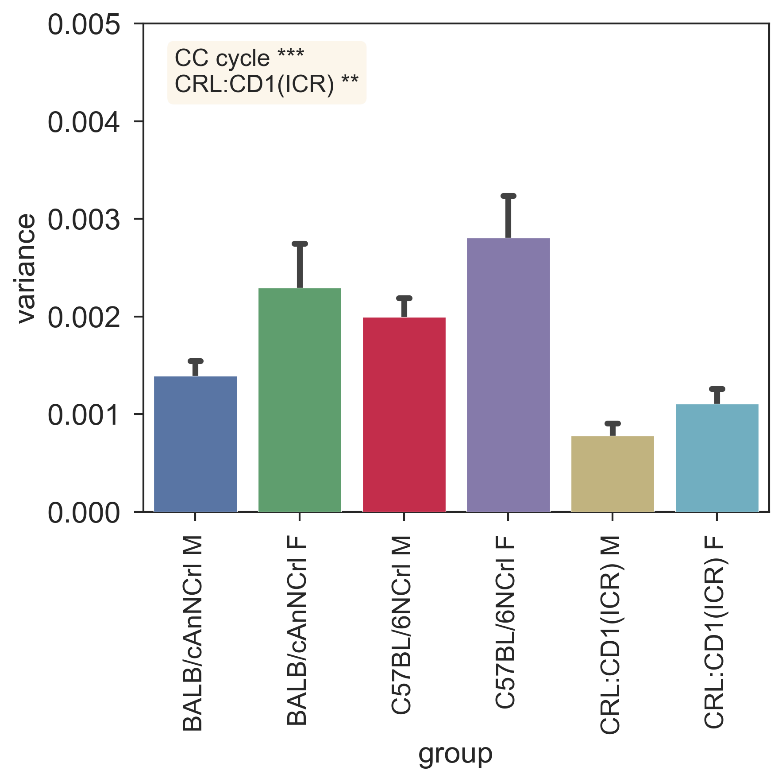


Supplementary Figure 1. **Spatial activity variance across the first hour of cage-change**. Average (± s.e.m.) Variance calculated over the activity values of the 12 electrodes. Significant fixed effects are reported (**p < 0.01, ***p < 0.001), with male C57BL/6NCRL used as reference (the model structure and relative statistical results are available in supplementary data materials).


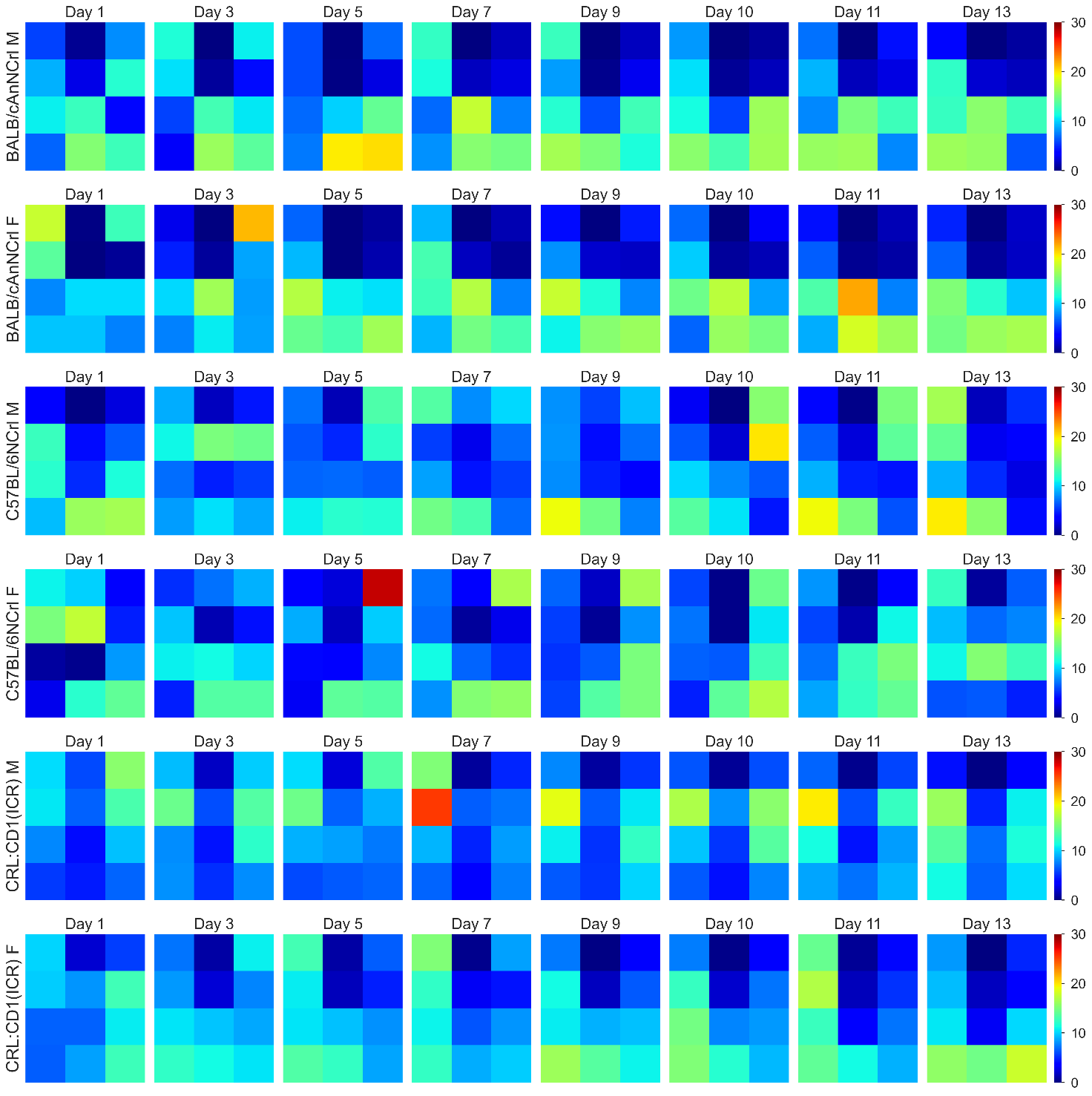


Supplementary Figure 2. **Spatial activity heatmaps during the lights-on phase across days of a cage-change cycle**. Average percentage of activity performed across the 12 electrodes (over the total activity performed on the DVC board), during the lights-on phase. The figure shows one example cage for each strain and sex, across multiple days of the first cage-change cycle. The rear half of the cage corresponds to the 6 electrodes in the top part of each heatmap, while the front half corresponds to the 6 bottom electrodes of each heatmap, as displayed in Figure 6.


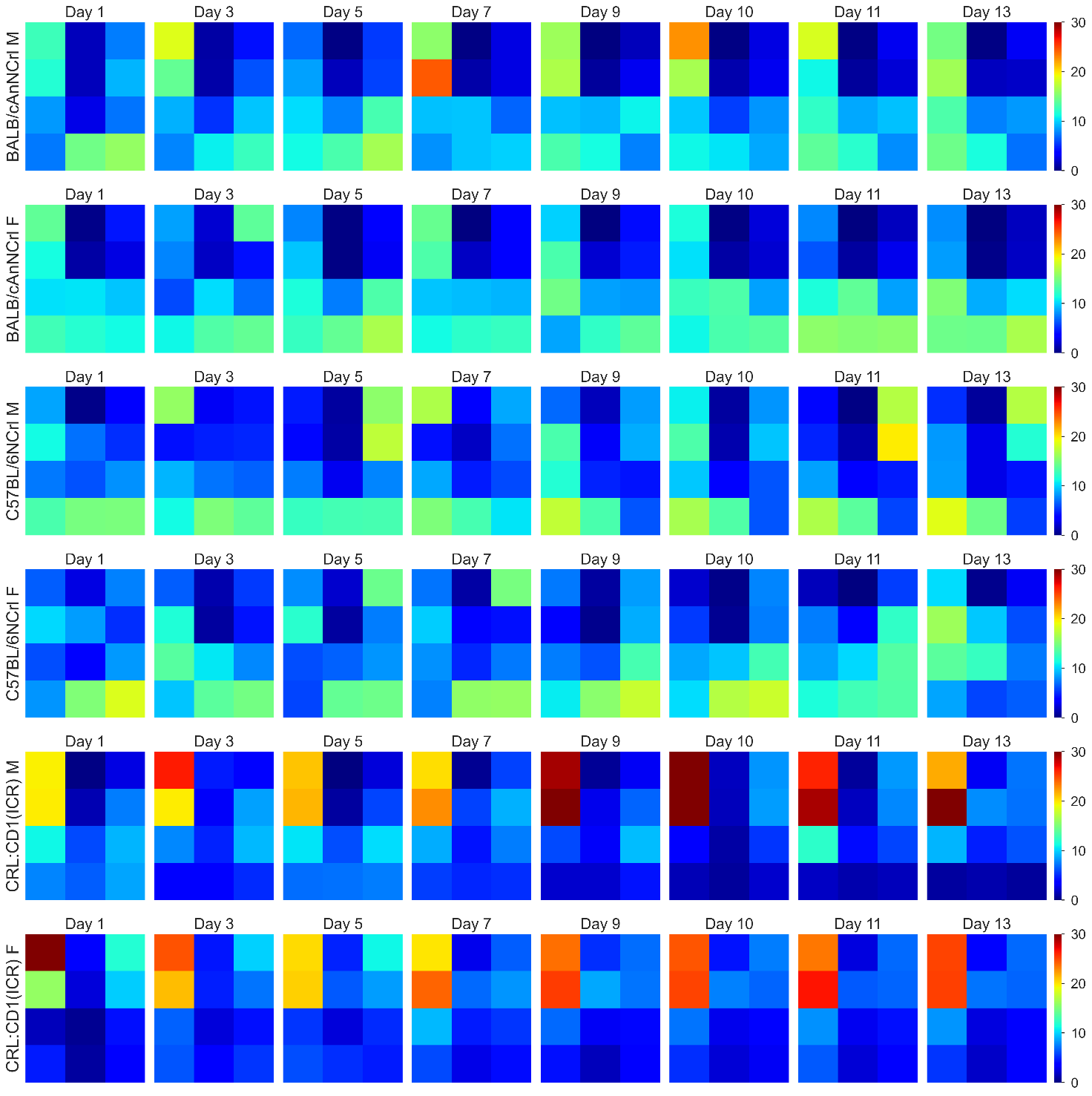


Supplementary Figure 3. **Spatial activity heatmaps during the lights-off phase across days of a cage-change cycle**. Average percentage of activity performed across the 12 electrodes (over the total activity performed on the DVC board), during the lights-off phase. The figure shows one example cage for each strain and sex, across multiple days of the first cage-change cycle. The rear half of the cage corresponds to the 6 electrodes in the top part of each heatmap, while the front half corresponds to the 6 bottom electrodes of each heatmap, as displayed in Figure 6.


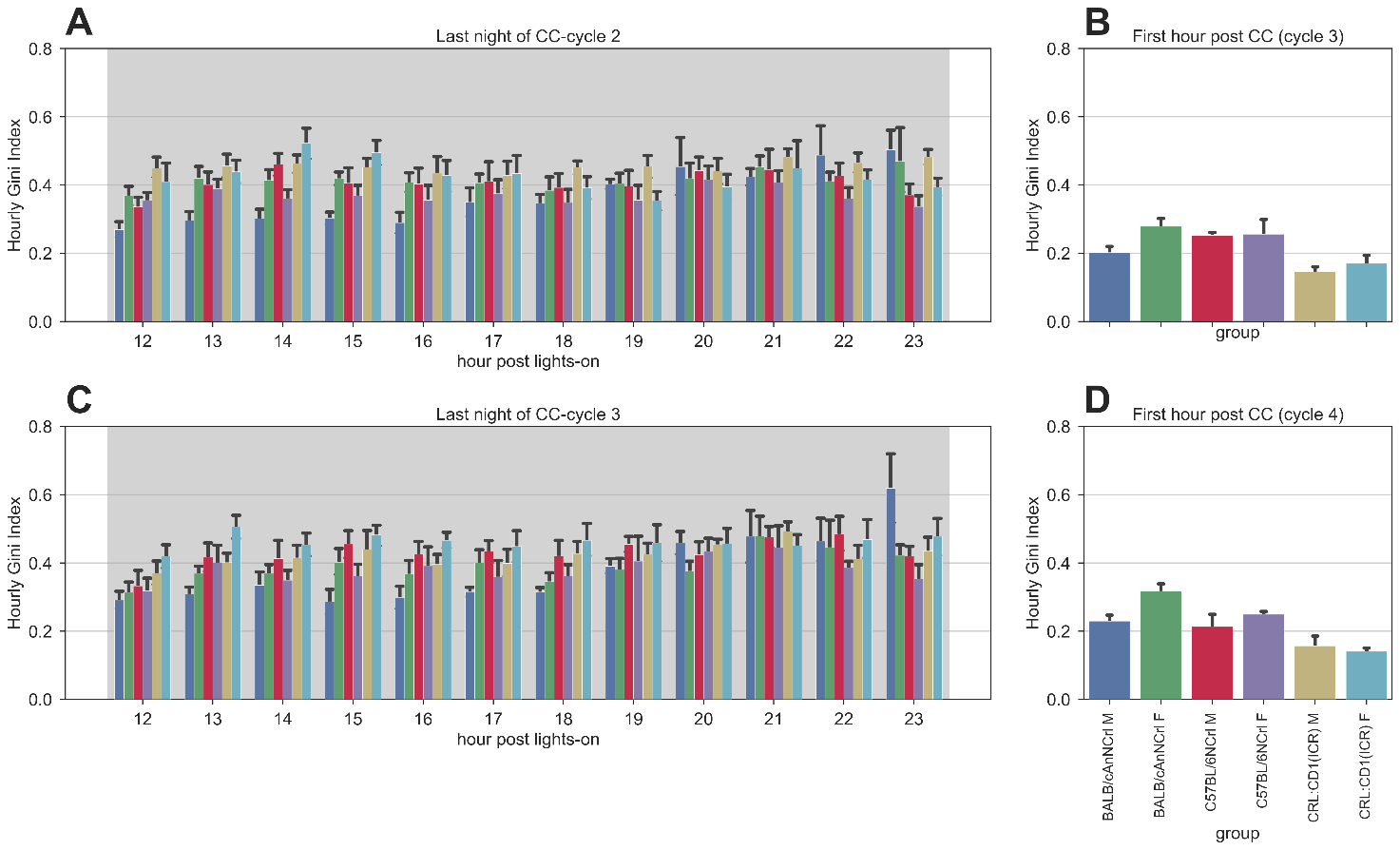


Supplementary Figure 4. **Gini Index in-between the cage-change interval**. **A.** Average (± s.e.m.) Gini Index of activity values of the 12 electrodes calculated for each hour of the night previous the cage-change, during the second cage-change cycle. **B.** Average (± s.e.m.) Gini Index of activity values of the 12 electrodes of the first hour of the third cage-change, following the night in 4A. **C.** Average (± s.e.m.) Gini Index of activity values of the 12 electrodes calculated for each hour of the night previous the cage-change, during the third cage-change cycle. **D.** Average (± s.e.m.) Gini Index of activity values of the 12 electrodes of the first hour of the fourth and last cage-change, following the night in 4C.
